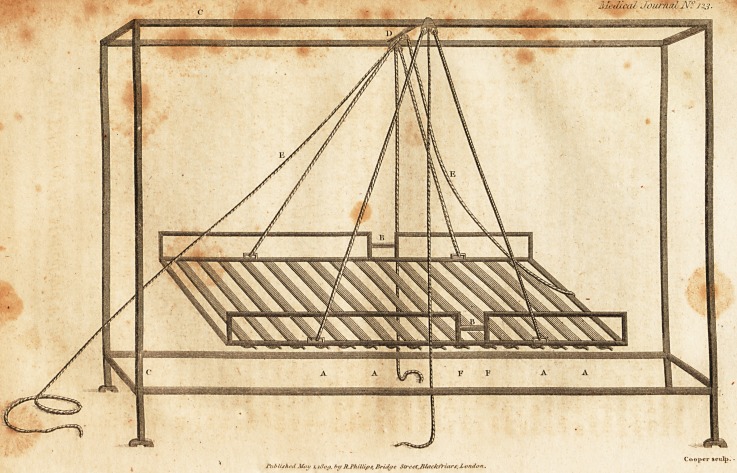# On the Use of Stimulants in Burns

**Published:** 1809-05

**Authors:** 


### *To the Editors of the Medical and Phyſical Journal.*

Gentlemen,

THE frequent fatality of extensive burns on the body, and some distressing circumstances of this kind having fallen within the circle of my observation, have induced me to offer a few remarks on this subject, through the extensive medium of your very valuable Journal.

If we duly consider the action of fire on the living fibre, if we attentively observe the outlines of that action, and the dreadful inroads it makes on the constitution, we shall not be surprised at its fatal effects, when applied in an extensive degree. A long experience has pointed out to us many valuable remedies to counteract this fatal termination; but as there are circumstances which certainly are improper to be treated as they sometimes are, I shall in this sheet point out in as clear a manner as I can, the propriety and impropriety of the treatment of those circumstances.

When fire is applied to the living body, a burning pain is produced, the parts immediately under its influence are deprived of their vitality, the vessels are destroyed, its effects are extended deeper and wider in a direct ratio to the continuance of the fire to the part, and the pain to a certain degree is increased in proportion to its continuance. I say to a certain degree only, because when the sentient vessels are destroyed, or their sensibility or irritability annihilated by the excess of stimulus, the nerve will no longer be susceptible of sensation at that immediate part, and therefore the pain will not be augmented; but the lesion and destruction of the soft or hard parts will be extended as long as the cause exists. These are the effects of fire when applied in a considerable degree. Let us now consider its action extended to a wider sphere, as when a person's clothes take fire: here we see a large surface on the body suffering under its ravages, involving perhaps the whole parietes of the abdomen and thorax, or the whole of the back in its circle. Now, if we observe what happens, we shall find that a person so burnt will not complain much of the pain, but his principal complaint will be, of the immense fatigue he feels, of his strong inclination to sleep, and he will very much wish to indulge in quietude and repose; if the pulse be felt, it will be found to be quick and weakish, and in a few hours delirium will most commonly supervene.

Cooper sculp.

Published May 1, 1809, by R. Phillips, Bridge Street, Blackfriars, London.

supervene. In the principal circumstances with regard to the effect, intense cold acts as severe as burns, for both subdue the powers of life, and both incline the person to sleep. It is a law of the animal economy, that the greater the excitement the greater will be the reduction of the vigor. On this law we must argue, that so powerful a stimulus as we are supposing, must be followed by a very considerable reduction of the powers of life, and therefore it is that we see those who have been very much burnt, sink so very rapidly under its effects.

When a person has suffered considerably from fire, and especially if it be about the abdomen or the trunk, to any considerable extent, we are to adopt our measures of relief as soon as possible, for the timely practice of them will very powerfully tend to rescue the person from that destruction which will very probably follow. Now, cold has been very generally resorted to in those cases, to diminish the pain, and to lessen the action of the fire; it may be sometimes proper, but I believe it is more frequently improper than otherwise, where the burn is so extensive as we suppose. For, if we carefully consider the operation of cold, we shall find it to be a very powerful sedative, and therefore if applied when the body has been much burnt, and the powers of life consequently much subdued, its action will be followed by the worst of consequences, and it will most probably hasten that termination which it was intended to counteract. For if the person be extensively burnt the vital actions will soon become languid, and if cold be now applied, the consequences which will happen I need not describe.

I therefore propose, that in all cases, where more than a quarter of an hour has elapsed, and where the person has been so much burnt as to make him feel only a small proportion of pain, in comparison to the extent of the burn, and at the same time feels very weak, and is disposed to sleep, he be supported by the most nutritious diet; that cordials be instantly given; and that the powers of life be raised by those means being very diligently used. Now, having revived the patient from the subdued state in which he was, the warm bath may be employed for a few minutes to favour the circulation through the system; and this being done, the surface of the body may be covered with cloths wet with ol. lini. & aq. calcis. If he be so much burnt as not to admit of being handled without danger of detaching the flesh from the body, the warm bath had better be omitted; and as soon as the powers of life have been recalled for a few hours, I should advise a purgative of calomel and rhubarb to clear the bowels, in order to give the medicines to be afterwards exhibited, the power of producing their full effects. In this manner the cure should be conducted, unless high inflammatory symptoms supervening should contraindicate. If, however, the patient be of an inflammatory diathesis; the burn not so extensive; the patient very sensible to the pain; if the pulse be quick and hard, and he be very thirsty, and very restless, the cold bath should be used; the antiphlogistic regimen should be adhered to in its fullest extent, and the same applications as in the last case, viz. ol. sem. lini & aq. calcis. If, however, the patient be of a phlogistic diathesis, and at the same time nervous, a middle course should be steered, the worst symptoms attacked by their appropriate remedies, and a due balance in the system preserved. Opiates may be exhibited where the irritation is considerable, and when the destroyed surface begins to discharge and slough off, a mild poultice of bread and milk will be useful. As there is not any machine within my observation, whereby a person much burnt may be raised to have his dressings applied, or to be raised from his bed conveniently, I have subjoined the rough sketch of a plan which may become a great source of relief to the unhappy sufferer.

I am, &c.

PHILANTHROPUS.

### EXPLANATION OF THE PLATE.

The large frame marked C is a bedstead, D is a bar across the top, to which may be attached a tackle for the rope E to slide through. Suspended from the bedstead is a cradle, the bottom of which is composed of girths, with buckles at the side marked A; these may be undone, in order to get to any part of the body, posteriorly, when the cradle is raised by the ropes; the frame is six feet in length, $3\frac{1}{2}$ of which is at the lower, and $2\frac{1}{2}$ at the upper part; at the division is a hinge on each side, to allow of the lesser portion being raised to a right angle, which is done by the rope E being pulled; the width of the cradle is 2 feet; on each side of the cradle is seen a rail of about 6 inches high, of iron, or any thing capable of resisting the patient rolling out when the cradle is raised; a small bar of iron marked B is fixed to keep the cradle extended when it is wished to raise it, in order that the patient may have his bed made; when it is wished to raise the upper portion of the cradle to a right angle, the bars of iron B on each side may be taken out, and all the railing may be taken out when the patient is on the bed. When the patient is to be dressed on the back, the upper upper part is raised at right angles, whereby the patient is made to sit, and the girths being unbuckled, the whole of the back may be exposed and dressed; when the parts about the nates are burnt, a rope may be attached to the inferior portion, as is seen at the superior portion, so that the lower part may be raised in like manner, by fixing a hinge on each side at F; two ropes are seen attached on each side, and united at the top, passing through a tackle; the rope to be pulled runs down in the middle; by this contrivance two persons only are required to do every thing relative to the patient's comfort; this of course may be used for the bed-ridden.

## Figures and Tables

**Figure f1:**